# TGF-β/Smad3 Signalling Modulates GABA Neurotransmission: Implications in Parkinson’s Disease

**DOI:** 10.3390/ijms21020590

**Published:** 2020-01-16

**Authors:** Mª Dolores Muñoz, Nerea de la Fuente, Amelia Sánchez-Capelo

**Affiliations:** 1 Unidad de Neurología Experimental, Hospital Universitario Ramón y Cajal—IRYCIS, ES-28034 Madrid, Spain; domuara@gmail.com; 2 Servicio de Neurobiología-Investigación, Hospital Universitario Ramón y Cajal—IRYCIS, ES-28034 Madrid, Spain; nereafb_14@hotmail.com

**Keywords:** GABA, Parkinson’s disease, TGF-beta, Smad3, dopamine, synaptic plasticity, cognition

## Abstract

γ-Aminobutiryc acid (GABA) is found extensively in different brain nuclei, including parts involved in Parkinson’s disease (PD), such as the basal ganglia and hippocampus. In PD and in different models of the disorder, an increase in GABA neurotransmission is observed and may promote bradykinesia or L-Dopa-induced side-effects. In addition, proteins involved in GABA_A_ receptor (GABA_A_R) trafficking, such as GABARAP, Trak1 or PAELR, may participate in the aetiology of the disease. TGF-β/Smad3 signalling has been associated with several pathological features of PD, such as dopaminergic neurodegeneration; reduction of dopaminergic axons and dendrites; and α-synuclein aggregation. Moreover, TGF-β/Smad3 intracellular signalling was recently shown to modulate GABA neurotransmission in the context of parkinsonism and cognitive alterations. This review provides a summary of GABA neurotransmission and TGF-β signalling; their implications in PD; and the regulation of GABA neurotransmission by TGF-β/Smad3. There appear to be new possibilities to develop therapeutic approaches for the treatment of PD using GABA modulators.

## 1. Introduction

In the central nervous system (CNS), the most widespread inhibitory neurotransmitter is γ-aminobutiryc acid (GABA). At inhibitory synapses, GABA is involved in controlling the neuron’s excitatory/inhibitory balance and in synchronizing neuronal networks to produce the oscillations implicated in cognitive processes [1,2]. The basal ganglia are a group of brain nuclei involved in movement, cognition and mood, and they are mainly composed of GABAergic neurons. Parkinson’s disease (PD) is known to be associated with general alterations to the basal ganglia nuclei, although altered GABA neurotransmission may also arise in other brain regions in PD patients, such as the hippocampus or cortex. Such changes would influence synaptic plasticity and cognitive processes, such as motor learning or sensorimotor processing. However, the role of GABA neurotransmission in PD is still not fully understood. GABA receptors’ (GABARs) trafficking to the plasma membrane is highly regulated to control the strength of synaptic inhibition. Variation in the distribution of postsynaptic GABARs may alter the equilibrium between excitatory and inhibitory neurotransmission, and may be associated with several CNS pathologies, such as epilepsy, ischemia, PD and Alzheimer’s disease (AD) [3].

Deficiencies in the transforming growth factor β (TGF-β) intracellular signalling pathway have been associated to an increased catabolism of striatal dopamine (DA), postnatal neurodegeneration of midbrain dopaminergic neurons, the formation of α-synuclein deposits and motor and cognitive alterations. Indeed, deficiencies in TGF-β signalling increase GABA neurotransmission in the midbrain and the hippocampus [4]. This review focuses on recent research showing how GABA neurotransmission can be modulated by TGF-β signalling. Further comprehension of GABA neurotransmission in the context of PD and its modulation by TGF-β may provide new insights into the molecular mechanism involved in motor and cognitive alterations of the disease. Indeed, new pharmacological targets may be identified, and modulators of GABA neurotransmission may be evaluated for the treatment of PD.

## 2. GABA Neurotransmission

### 2.1. GABA Biosynthesis and Turnover

The inhibitory neurotransmitter GABA is present in interneurons across the brain, in which it is produced by the decarboxylation of cytosolic glutamate by glutamic acid decarboxylase (GAD) [1]. In turn, this glutamate is produced by the hydrolytic action of the glutaminase enzyme acting on glutamine. In addition to its neurotransmitter role, GABA can be generated in the tricarboxylic acid (TCA) cycle through what is called “the GABA shunt,” whereby adenosine triphosphate (ATP) is produced in the mitochondria and participates in energy homeostasis. GAD is the rate-limiting enzyme in GABA biosynthesis, and it is found in two cytosolic forms, GAD65 and GAD67, with molecular weights of 65 and 67 KDa, respectively. These enzymes have distinct intracellular distributions, and while GAD65 is located in axon terminals and participates in GABA biosynthesis, GAD67 is present in the cell body and dendrites, and it may drive the metabolic effects of GABA [5]. GAD activity can be modulated by post-translational modifications, including phosphorylation, palmitoylation and activity-dependent cleavage [6]. While GABA biosynthesis occurs in neurons, its glutamine precursor is also present in astrocytes, which may be generated by the TCA cycle. As such, astrocytes surrounding GABAergic synapses are a probable source of the glutamine precursor [5].

Once GABA is released into the synaptic cleft, a proportion is transported to astrocytes (10–20%), while the rest is transported back to the presynaptic terminal and packaged into vesicles to be released again. Different GABA transporters have been identified, three with high affinity (GAT1, GAT2 and GAT3), and another with no specificity for GABA (betaine-GABA transporter, BGT1): GAT1 is expressed in neurons and astrocytes; GAT2 is expressed in the neonatal brain; GAT3 is mainly found in astrocytes; and BGT1 is scarcely expressed [7]. Inactivation of GABA neurotransmission is mediated by enzymatic degradation of GABA in the mitochondria of neurons or astrocytes, a process mediated by the enzymes GABA-transaminase (GABA-T) and succinic semialdehyde dehydrogenase (SSADH) [8].

### 2.2. Synaptic and Non-Synaptic GABA Neurotransmission

GABAergic neurons communicate through both synaptic (phasic) and non-synaptic (tonic) transmission, with GABA released from synaptic vesicles producing transient or phasic inhibition, while low concentrations of ambient GABA produce tonic inhibition. Phasic inhibition mainly controls neuronal excitability and prevents over excitation of neurons, but also generates rhythmic oscillations in neuronal networks by phasing and synchronizing the activity of neuronal populations. Tonic inhibition can induce slow regulation of the membrane conductance and potential. However, GABA release from multiple vesicles or from several terminals can induce GABA spill over, resulting in the activation of both phasic and tonic transmission [9]. Indeed, lateral receptor diffusion in the postsynaptic neuron allows a rapid interchange between extrasynaptic and synaptic locations [10].

GABA is released into the synaptic cleft by depolarization of presynaptic neurons, where it can target postsynaptic GABARs. GABARs can be classified as GABA_A_ and GABA_B_ receptors (a third GABA_C_ receptor was recently reclassified as GABA_A_). GABA_A_ receptors (GABA_A_Rs) represent the most abundant inhibitory receptor and mediate fast synaptic transmission. These receptors are heteropentameric arrangements that combine elements from 19 subunit classes (α1-6, β1-3, γ1-3, δ, ε, θ and π). They are normally composed of two α, two β and one γ subunit arranged in a α–β–α–β–γ manner. GABA_A_Rs are mainly found in postsynaptic membranes, but they may also concentrate at presynaptic sites, and extra-synaptically in the membranes of dendrites. The α1-3, β1-3 and γ2 subunits are normally integrated into synaptic GABA_A_Rs, whereas the α4-6 and δ subunits are the main components of extra-synaptic GABA_A_Rs [11]. The two αβ interfaces form extracellular GABA binding sites, while the αγ interface generates a site for benzodiazepines binding. Benzodiazepines are small molecules that target GABA_A_Rs, acting as positive allosteric modulators that are commonly used clinically to manage anxiety disorders [12]. GABA_A_Rs constitute inhibitory chloride ion channels that, upon activation, introduce chloride and bicarbonate ions into the postsynaptic neuron to mediate inhibitory currents. Their localization is regulated by their subunit composition, which also influences the channel’s properties and pharmacological sensitivity [13]. Alternatively, GABA_B_Rs are heterodimeric metabotropic receptors coupled to multiple ionic currents by G-proteins [14]. GABA_B_Rs have a higher sensitivity to GABA than GABA_A_Rs, yet with a slower time-course that can respond effectively to low concentrations of extra-synaptic GABA [15].

At rest, the inside of the neuron is negatively charged relative to the outside, and neuronal permeability to chloride ions through GABARs enhances the net inward flow of anions. This increments the negative charge of the postsynaptic neuron and provokes hyperpolarization, commonly described as an inhibitory postsynaptic potential (IPSP). The effect of GABA is to reduce the probability of action potential initiation [9]. GABAergic transmission thereby modulates the strength and timing of postsynaptic spike outputs [16].

### 2.3. Postsynaptic Activation by GABARs

**Figure 1:**
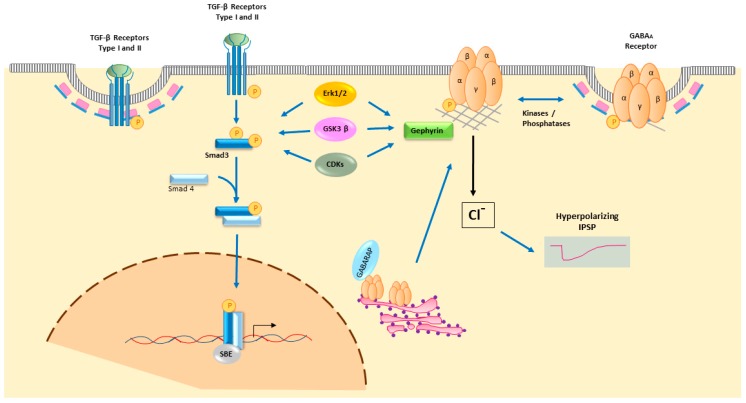
Summary illustration of TGF-β signaling and postsynaptic GABAR neurotransmission.

GABA_A_R trafficking plays a central role in receptor activity, and indeed, interactions of several scaffold and non-scaffold proteins with the receptor modulate inhibitory synapses. Once the GABA_A_R subunits are expressed and translated, they are assembled in the endoplasmic reticulum with the help of Plic-1, and transported to the Golgi apparatus, where they bind to GABARAP (γ-aminobutyric acid receptor-associated protein)/NSF (*N*-ethylmaleimide-sensitive factor) complexes. This complex is then palmitoylated by Golgi-specific DHHC (Asp-His-His-Cys) zinc finger protein (GODZ) to be transported to the plasma membrane. Other molecules involved in GABA_A_R delivery to the plasma membrane are Big2, GRIP and PRIP [17]. These processes of receptor clustering and trafficking to the cell surface define the availability of the receptor at both synaptic and extrasynaptic locations (Figure 1).

In addition, GABA_A_Rs may undergo endocytosis at the plasma membrane—mediated by clathrin and dynamin, and involving the AP2 adaptor molecule [18,19,20]. GABA_A_R internalization is modulated by the action of different protein kinases and phosphatases (e.g., PKA, PKC, calcineurin, CamKII, Src, Akt or PKC), controlling the receptor’s subcellular localization, its kinetics and its conductance. [20,21]. Once internalized, GABA_A_Rs can be stored until they are again required at the plasma membrane, or alternatively, they may be degraded by lysosomes. For their transport back to the plasma membrane, an interaction with HAP1 (huntingtin-associated protein) is required to avoid degradation [22]. Recently, interfering with GABA_A_R trafficking was proposed as an interesting way to modulate the inhibitory GABA pathway [23].

In order to concentrate GABA_A_Rs at the postsynaptic density (PSD), the scaffold protein gephyrin anchors the receptor subunits to the cytoskeleton. Gephyrin is a molecule on which different intracellular signalling pathways that phosphorylate this protein converge, such as the Erk1/2, GSK3β, CDKs or nNOS signalling pathways. Moreover, gephyrin interacts with different molecules to modulate synapse formation and plasticity. These include neuroligin2, a cell adhesion molecule that can interact with presynaptic neurexin and favour crosstalk between pre- and post-synaptic neurons at synapses [23,24,25]. By contrast, the concentration of extrasynaptic GABA_A_Rs is mediated by anchoring the receptor to phosphorylated radixin, which links actin to the plasma membrane to concentrate channel activity beyond the synaptic terminal [26,27].

## 3. GABA in PD

PD is a slowly-progressing neurodegenerative disorder characterized by motor (resting tremor, rigidity, bradykinesia and postural instability) and non-motor symptoms (dementia, sleep disorders, depression, orthostatic hypotension, oesophageal and lower bowel dysmotility, urinary alterations, anosmia and seborrheic dermatitis). Its histopathological hallmarks are nigrostriatal dopaminergic neuronal loss, with the consequent decrease in striatal DA release, and the intracytoplasmic aggregation of misfolded α-synuclein that forms Lewy bodies and neurites [4].

### 3.1. Striatal GABA Neurotransmission

The basal ganglia are a group of subcortical nuclei involved in movement, cognition and mood, and they are mainly composed of GABAergic neurons; i.e., the striatum (ST), globus pallidus (GP), subthalamic nucleus (STN) and the substantia nigra (SN) in the midbrain [28]. In general, the rodent ST can be divided in two main sub-regions: (a) the dorsal or neostriatum, which can be further sub-divided into the dorso-lateral and dorso-medial ST; and (b) the ventral ST, which includes the olfactory tubercule and the nucleus accumbens (NAcc). Functionally, the dorsal ST is involved in motor planning, habit learning and action selection, while the NAcc participates in reward and motivational behaviours. Different GABAergic neurons are located in the ST, mainly medium-sized spiny neurons (MSNs) that constitute 95% of the striatal neurons, while the remaining 5% are cholinergic or GABAergic interneurons [29].

MSNs are the main projection neurons of the ST and they are modulated differentially by DA. In addition to GABA expression, half of the MSNs express dopaminergic D1 receptors, and they project to the SN pars reticulata (SNpr; striatonigral neurons), the ventral tegmental area (VTA) and to the internal GP (GPi), forming the direct pathway of the basal ganglia. The other half of the MSNs express D2 receptors, and they project to the external GP (GPe) and the ventral pallidum (striatopallidal neurons), forming the indirect pathway. Another specific marker expressed by striatonigral GABAergic neurons is substance P, whereas striatopallidal GABAergic neurons express encephalin [30]. It has also been proposed that a small proportion of MSNs may co-express both D1 and D2 receptors, although their function remains unclear [31]. Classically, it has been considered that the direct pathway facilitates movement, while the indirect pathway inhibits movement. However, both pathways are more active when moving or performing a task than when at rest, and both are necessary to facilitate movement. Indeed, disrupting the equilibrium between both pathways may provoke several movement disorders. Recently, a different role for these pathways was suggested, indicating that they are implicated in the initiation of movement. Optogenetic manipulation of the striatonigral and striatopallidal pathways suggests that the direct pathway promotes the initiation and continuation of movement, and the indirect pathway suppresses other behaviours to allow movement to be performed, suggesting a coordinated rather than opposite activity for these pathways [32].

MSNs receive glutamatergic inputs from cortical and thalamic neurons, and DA innervation from the SN. Quiescent MSNs have a very hyperpolarized membrane potential and excitatory glutamate inputs induce MSN firing. DA is thought to modulate neuronal excitability to raise the signal-to-noise ratio acutely by activating both D1 and D2 dopamine receptors in MSNs [29,30]. Short-term corticostriatal and thalamostriatal glutamatergic synaptic stimulation evokes distinct patterns of MSN spiking, with cortical synapses facilitating, and thalamic synapsis depressing, post-synaptic depolarization [33]. This differential regulation of MSNs by glutamate may be involved in suppressing an ongoing movement when attention is required [34]. In addition to the excitatory glutamatergic projections from the cortex, two distinct corticostriatal GABAergic projections that innervate the dorsal ST have also been identified, originating in the primary (M1) and secondary (M2) motor cortex, and modulating motor activity distinctly [35].

The interaction of DA with the ST is complex, considering the variety of D1-like (D1 and D5) and D2-like (D2, D3, and D4) receptors present in MSNs and interneurons. Indeed, nigral DA neurons can also release GABA to the dorsal ST, although GABA is not synthesized through the canonical GAD65 and GAD67 enzymes, but rather, via a non-canonical aldehyde dehydrogenase 1a1 pathway [36]. In addition, GABA can be taken up through plasma membrane in midbrain DA neurons [37]. Further interactions between DA and GABA have been observed, since nigrostriatal DA release is inhibited by striatal GABA_A_R and GABA_B_R activation. Furthermore, endogenous striatal GABA produces tonic inhibition of DA release, mainly through GABA_B_R [38].

Striatal GABA may also come from auditory and motor cortex afferents to the dorsal ST [39]. In addition, reverse projection from the GP to the D1 MSNs (arkypallidal neurons), and to interneurons, may provide an important extrinsic source of GABA in the ST [40]. Another GABAergic projection identified is that from the bed nucleus of the stria terminalis to the patch compartment of the dorsal ST [41].

Besides to the striatopallidal and striatonigral projections, MSNs extend collateral branches and their axons form GABAergic synapses with neighbouring cells. This extensive intrastriatal axonal arborisation induces collateral inhibition that is mediated by postsynaptic GABA_A_R, also known as surround inhibition [42]. Although collateral MSN connections can couple striatonigral and striatopallidal MSNs, D1 MSNs mainly form functional connections with other D1 MSNs, whereas D2 MSNs connect with both D1 and D2 MSNs. Indeed, there are significantly fewer GABA_A_Rs in D1 than in D2 MSNs, suggesting a weaker functional significance of GABAergic collateral inhibition in striatonigral projections [43]. A molecular mechanism implicated in collateral inhibition involves nitric oxide (NO), which increases the expression of the vesicular GABA transporter (VGAT), which in turn modulates GABAergic signalling in local MSN collaterals [44].

Interneurons are the other GABAergic neurons in the striatum. Although they do not express D2 receptors, they have spiking properties that differ from those of MSNs, and they are very rare [45]. This group of neurons is very heterogeneous and can be classified as fast-spiking (FSI), low-threshold spiking (LTSI) and calretinin (CR) interneurons. FSI neurons express parvalbumin, while LTSI neurons express somatostatin, nitric oxide synthase (NOS) and neuropeptide Y [29]. Other subpopulations of GABAergic interneurons may express tyrosine hydroxylase (TH), the rate-limiting enzyme of DA biosynthesis, although they do not release DA and they are further classified into four subtypes [46]. Differential combinations of DA receptors are present in all types of interneuron, suggesting further modulation of the local striatal circuitry by DA. In summary, striatal interneurons and collateral interactions of MSNs form a local circuit that is considered to compute motor, limbic and sensory information into cognitive or behavioural outputs [29].

### 3.2. Striatal GABA Neurotransmission in PD

Early studies showed enhanced GAD65 and GAD67 expression in different models of PD, such as rats treated with 6-OHDA [47] or monkeys treated with MPTP. The GAD65 and GAD67 enzymes are present in monkeys, and they co-localize in most striatal MSNs, both in the caudate nucleus and putamen. Induction of parkinsonism with MPTP increases GAD65 and GAD67 gene expression in preproenkephalin-labelled neurons and striatopallidal neurons, and this effect is not observed in striatonigral neurons. This increment is probably associated to enhanced GABAergic activity in striatopallidal neurons and GABA release in the GP [48]. Indeed, a new model of bradykinesia has been developed by selective deletion of D2 receptors from the indirect MSN pathway. Striatopallidal D2 depletion does not alter DA transmission, but rather, it leads to enhanced GABAergic tone, and decreased striatal and pallidal neuron firing, which may provoke motor dysfunctions that are related to bradykinesia [49]. GABA is also elevated in the pons and putamen of patients with mild-to-moderate PD, although only with a mild change [50,51]. Excessive exposure to manganese is an environmental factor that reflects a high risk of developing PD, increasing GABA levels in the putamen, and in other brain regions, such as the thalamus and GP [52]. Indeed, in a unilateral 6-OHDA model of PD, GABA content increases in the striatum [53]. However, GABA_A_R binding analyses have not found different levels in the putamen and caudate of MPTP-treated monkeys, or in post-mortem human brains [54,55].

In situations of DA depletion, striatal neurons try to compensate this loss by adapting intrinsic excitability and synaptic plasticity in MSNs. As such, the loss of D1 MSN signalling induces increased intrinsic compensatory excitability, whereas the loss of D2 MSNs reduces intrinsic excitability [56]. This compensation delays the onset of motor symptoms, but in later stages of the disease, the alterations to MSNs induce an imbalance between the direct and indirect pathways that could be central to the hypokinetic symptoms of PD. In PD, a decrease in DA reduces the activation of D2 receptors, and it leads to weaker GPe activation and hyperactivity in the indirect pathway, resulting in increased inhibition of thalamic neurons and decreased excitation in the cerebral cortex [28].

The effect of DA denervation on striatal MSN collateral inhibition is not clear, and a study using reserpine or unilateral 6-OHDA injection showed nigrostriatal dopaminergic depletion to strongly reduce GABAergic connections between both D1 and D2 MSNs [57]. However, in a study of Pitx3^-/-^ mice that suffered selective and severe DA neuron loss in the SN, and DA denervation in the dorsal ST, axon collateral connections to the dorsal ST were not disrupted after DA denervation [45]. Further studies will be necessary to clarify the role of surround inhibition in the parkinsonian ST, although these local axon collaterals may help shape the striatal output [29].

### 3.3. Nigral GABA Neurotransmission in PD

The cell bodies of DAergic neurons in the SN are located in the pars compacta (SNpc), which lies dorsally to the SNpr, where the dendritic projections of DAergic neurons interact with GABAergic neurons—the main neuronal component of the SNpr. DAergic neurons fire spontaneously, with half of the neurons firing in a random mode, one third firing with regular pacemaker activity and 15% with a slow burst pattern [58]. These firing patterns are stimulated by glutamatergic inputs and modulated by GABA_A_Rs [59]. The main afferents of DAergic neurons in the SNpc are GABAergic neurons that arise from the neostriatum, the GPe or SNpr, providing local interactions between DA and GABA that are relevant for the function of nigral DAergic neurons [58]. Striatonigral MSNs also project to the SNpr, and express high levels of D1 receptors in their terminals [60]. Other GABAergic afferents to DAergic neurons in the SN arise from the superior colliculus, lateral habenula, peripheral nociceptive stimulation and the central nucleus of the amygdala [61]. GABAergic inputs from both afferent neurons and collateral SNpr GABAergic neurons modulate DAergic neurons. Indeed, DA released from the dendrites of SNpc neurons activates presynaptic D1 receptor of MSNs to facilitate GABA release via the cAMP pathway, reinforcing the interplay between both systems [62].

**Figure 2:**
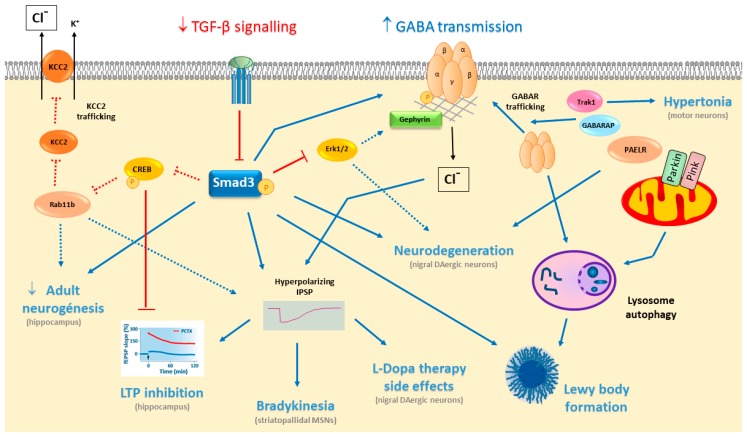
Illustration of the working model of TGF-β signaling and GABA neurotransmission interaction in the context of PD. Arrows indicate induction and T-bars inhibition. Dotted arrows and dotted T-bars suggest possible interactions, not yet shown experimentally and derived from the bibliographic analysis.

Somatodendrites of DAergic neurons have GABA_A_Rs and GABA_B_Rs, which induce hyperpolarizing IPSPs and inhibit spontaneous DAergic activity by increasing Cl^–^ ion conductance through GABA_A_R stimulation, or by increasing K^+^ conductance following GABA_B_R stimulation [63,64]. Optogenetic studies have shown that DA denervation increases GABA release from striatonigral MSNs projecting neurons in the SNpr of 6-OHDA hemiparkinsonian mice. This increase enhances inhibition and changes the firing patterns of the SNpr, which may influence the supersensitivity of the DA receptors observed after L-Dopa treatment. Indeed, a combined treatment with GABA modulators has been proposed to control L-Dopa-induced side-effects (Figure 2) [65].

### 3.4. Dysfunctional GABA_A_R Trafficking in PD

It has been proposed that altered GABA_A_R trafficking may be associated with PD through different mechanisms (Figure 2). Hypertonia, in reference to muscle spasticity and rigidity, is a motor symptom of PD, yet it is also evident in other neurological disorders, such as cerebral palsy, epilepsy or dystonia [66]. Mutations in the trafficking kinesin binding 1 protein (Trak1) in mice induce severe hypertonia and a strong decrease in GABA_A_R, mainly in motor neurons [67], probably due to direct interaction of Trak1 with GABA_A_R and the dysregulation of its endocytic trafficking [17].

GABARAP is involved in vesicle trafficking of GABA_A_R to the plasma membrane, yet like LC3, it is also a homologue of the mammalian autophagy-related gene (Atg) 8. Autophagy may be involved in GABA_A_R clearance [68], and both GABARAP and LC3 have been detected in Lewy bodies of patients with PD, and those with dementia with Lewy bodies (DLB), the latter a disorder related to PD but with more severe and earlier cognitive impairment. Indeed, there is a loss of GABARAP in the cerebral cortex of DLB patients [69]. Although these data require further validation (i.e., using different antibodies and double staining with Lewy body markers) they suggest that GABA signalling may be impaired in the cerebral cortex of PD and DLB patients.

Parkin is a molecule involved in mitochondrial homeostasis that is mutated in autosomal recessive forms of PD. Parkin ubiquitinates damaged mitochondria to promote their clearance by autophagy, a process known as mitophagy. Parkin mutations reduce the capacity to eliminate damaged mitochondria, stimulating their accumulation and leading to early-onset PD [70]. Parkin binds to the parkin-associated endothelin-like receptor (PAELR), leading to its ubiquitination and degradation. Mutations in the parkin gene induce PAELR aggregation in the endoplasmic reticulum, neurotoxicity and cell death. Recently, GABARAP was seen to bind to PAELR [71], which is also localized to Lewy bodies and Lewy neurites of PD patients [72]. Thus, alterations to these proteins might influence GABA_A_R trafficking, although further research is required to better define the exact processes involved.

## 4. TGF-β Signalling

### 4.1. Secreted TGF-β Ligands

TGF-β is a large family of secreted growth factors that play central roles in embryonic development (e.g., dorso-ventral patterning, left-right asymmetry, neural and neuronal differentiation and mesoderm induction) and in mature tissues (extracellular matrix—ECM, epithelial-mesenchymal transition, stem-cell renewal, bone and cartilage formation, haematopoiesis, the immune system and the brain). TGF-β regulates many different cellular processes in a context-dependent manner, including proliferation, differentiation, motility, adhesion, metabolism and cell death. Dysregulation of TGF-β is involved in the development of many diseases, such as cardiovascular disorders, cancer and metastasis, osteoarthritis, fibrosis and neurodegenerative disorders such as PD and AD [73,74,75].

The members of the TGF-β family are encoded by 33 genes in mammals. These proteins are homo- and heterodimers, and the family is comprised of TGF-βs (TGF-β1, TGF-β2 and TGF-β3), activins, nodal, BMPs, GDFs, myostatin, MIS and lefty molecules. In this review we will focus on the TGF-β subfamily, and primarily, the TGF-β1 homodimer. These factors are encoded as precursor proteins with three domains: a signal peptide that is removed during secretion to the extracellular space; a large precursor segment; the latent associated protein (LAP); and the mature carboxy-terminal protein. After secretion, the LAP is non-covalently associated to the mature TGF-β to maintain it in a latent form. This LAP contains a motif that recognizes integrins, promoting the formation of a complex between integrins and LAP-TGF-β. In addition, LAP-TGF-β can covalently bind to the large latent TGF-β-binding protein (LTBP), forming a large latent complex that is retained in the ECM, where it is stored for local and rapid activation of TGF-β when needed [76]. Remodelling of the ECM promotes the activation of latent TGF-β, as do thrombospondin, fibronectin, integrins, proteases or mutations in ECM proteins, such as fibrillins. In this way, TGF-β acts locally, as its activation is highly regulated and it does not diffuse [77]. Moreover, paracrine actions can be provoked by cytoneme-associated ligand presentation [78]. Mature TGF-β1, TG-β2 and TGF-β3 isoforms are highly conserved, and they have nine cysteine residues that can establish inter- and intramolecular disulphide bonds [79]. Similarities in the three-dimensional topology of these proteins suggest an ancient structural conservation between TGF-β and other growth factors (e.g., NGF, PDGF and GDNF) [77,80].

### 4.2. TGF-β Receptor Activation

Active dimeric TGF-β ligands bind to cell surface receptor complexes that are comprised of two type II and two type I receptors. In total five type II and seven type I receptors have been described, with both serine/threonine and tyrosine kinase properties. TGF-β1/-β2/-β3 type I receptors are TβRI (or ALK5), ALK1 and ALK2, and the type II receptor is TβRII. After their expression, translation in the endoplasmic reticulum and further post-translational modification in the Golgi apparatus, receptors are transported to micro-domains at the cell surface, and targeted to both clathrin-associated endosomes and caveolin lipid raft compartments (Figure 1) [81,82].

When the TGF-β ligand approaches the cell surface, it binds to the TβRII dimer and induces a conformational change that enables serine/threonine phosphorylation of the TβRI. This in turn induces a conformational change in TβRI that liberates FKBP12 from a GS domain and activates the kinase activity of the receptor. Smad7 is an inhibitory molecule that inactivates the GS domain of TβRI [83]. Co-receptors, such as betaglycan and endoglin, aid the ligand’s binding to the receptor. However, the ectodomain of betaglycan and endoglin can be cleaved and released to the extracellular space, favouring the sequestering of TGF-β and dampening its responsiveness [77].

Receptors are normally retained inside the cell in pools or reservoirs, and their availability at the cell surface heightens or dampens TGF-β responsiveness. The transmembrane metalloprotease TACE, also known as ADAM17, cleaves TβRI in response to Erk and p38 MAPK signalling, limiting TGF-β responses without decreasing ligand binding to TβRII. TβRI is then further cleaved by γ-secretase to release the TβRI cytoplasmic domain, which translocates to the nucleus to control the transcription of targeted genes. Further regulation of the receptor occurs through ubiquitinylation [77]. Another interesting mechanism of receptor availability is mediated by insulin and glucose through the activation of Akt intracellular signalling. Insulin and glucose activate Akt, which phosphorylates RabGAP, promoting cell surface trafficking of both TβRI and TβRII, and increasing TGF-β responsiveness of the cell [84].

### 4.3. Smad2/3 Intracellular TGF-β Signalling

Receptor activation transmits signals from the plasma membrane to the nucleus mainly by Smad molecules, although non-Smad signalling may be also induced. Eight Smads are encoded by the mammalian genome. The TGF-β subfamily (TGF-β1, TGF-β2, TGF-β3, activins, nodal and some GDFs) activates Smad2 and Smad3, whereas the BMP subfamily and some GDFs activate Smad1, Smad5 and Smad8. Finally, the inhibitory Smad7 and Smad6 regulate TGF-β and BMP subfamily signalling, respectively. Focussing on the TGF-β subfamily, after ligand binding, activation of TβRI recruits Smad2 and/or Smad3 in order to transmit signals to the nucleus. The Smad anchor for receptor activation (SARA) membrane-anchor stabilizes the TβRI-Smad2/3 complex and favours Smad2/3 activation, which is mediated by phosphorylation of two carboxy-terminal serines. Following this phosphorylation, Smad2/3 dissociates from TβRI and binds to Smad4, promoting the translocation of the complex into the nucleus, where it will act as a transcription factor [85,86].

Smad2/3 proteins are formed by two conserved globular MH1 and MH2 domains, connected by a linker region. MH1 in the N-terminal domain can bind to DNA by recognizing Smad binding elements (SBEs), a CAGAC and related sequence motifs. Two splice variants of Smad2 have been detected: the common Smad2 that cannot bind to DNA, and the smaller Smad2β isoform that binds to DNA like Smad3 [87,88]. MH2 is the C-terminal domain that mediates protein–protein interactions with numerous regulatory and effector proteins, including TβRI, other Smads, histone modifiers, DNA-binding co-factors, etc. [75]. The linker region between MH1 and MH2 is variable, and it can be phosphorylated by different kinases (e.g., MAPKs, CDKs or GSK3β), thereby permitting Smad2/3 crosstalk with other signalling pathways. Indeed, the phosphorylation state of the linker may influence Smad2/3 translocation to the nucleus. Moreover, the two main sites for phosphorylation (the C-terminal and linker regions) can be further modulated by phosphatases, such as PPMA1 or SCP [77].

In the nucleus, Smad2/3-Smad4 complexes bind DNA to regulate gene transcription. Smad2/3 are weak transcription factors that directly target SBEs in the promoter regions of genes. However, they usually cooperate with high affinity transcription factors to either activate or repress gene expression, such as that of cJun in the AP1 complex, ATF3, FoXI, p53 and C/EBPβ. In this way, Smad2/3 transcriptional activity is also dependent on other signalling pathways activated in the cell, providing a further level of cell-context regulation to this pathway [79,89]. Other signalling pathways that cooperate with Smad2/3 at this level include the Wnt, Notch and Hedgehog signalling pathways [90]. It is estimated that several hundred genes are directly targeted by Smad2/3, including the inhibitory *Smad7*, *plasminogen activator inhibitor-1*, the *CDKN1A inhibitor of p21* and *FOXA2* [74,75,91]. Furthermore, genetic and epigenetic regulation of TGF-β signalling has also been observed. The expression of several non-coding RNAs, microRNAs (miRs) and long non-coding RNAs (lncRNAs), is under the control of TGF-β signalling, such as the miR-200 family and miR-205, which are downregulated by TGF-β [92]. Smad3 also promotes alternative RNA splicing by binding to primary transcripts or by repressing genes that regulate splicing [93,94]. Moreover, Smad2/3 can target nascent pre-mRNAs to promote their methylation and degradation, dampening the synthesis of the protein targeted. In this manner, extracellular TGF-β regulates the epitranscriptome to promote rapid cellular responses [95].

In addition to the canonical intracellular Smad2/3 signalling, TGF-β ligands can also transduce signals through Smad-independent pathways, such as the MAPK, mTOR or PI3K/AKT pathways. Indeed, these pathways and Smad2/3 can interact at different levels, and overall such crosstalk makes TGF-β an orchestrator of cell-context dependent responses [77,96].

## 5. TGF-β/Smad3 in PD

### 5.1. Deficient TGF-β/Smad3 Signalling in Parkinsonism

TGF-β signalling has been associated to several pathological characteristics of PD [4]. The extracellular growth factor TGF-β1 is up-regulated in striatal regions and in the ventricular cerebrospinal fluid of PD patients [97,98]. It is also up-regulated in other nervous system disorders, such as AD [99,100,101,102], amyotrophic lateral sclerosis [103], ischemia [104] and spinal cord injury [105]. In experimental animal models, chronic TGF-β1 overexpression may participate in the disease pathology [106,107,108,109], and deficiencies in TGF-β signalling may represent a risk factor for the development of some brain disorders [110,111,112,113,114,115]. Indeed, several genetic variants of the 5′ region of the *TGFB2* gene have been associated with PD [116]. During mammalian embryonic development, TGF-β3, but not TGF-β1, is necessary for the survival of midbrain dopaminergic neurons at perinatal stages [117]. Hence, while TGF-β3 appears to exert its effects on newborn neurons, TGF-β1 might have pathological effects in adults. The expressions of TGF-β1/-β2/-β3, TβRI and TβRII receptors, and Smad2, Smad3, Smad4 and Smad7, have been detected in both the SNs and STs of mice, with the exception of TGF-β3 and ALK1 in midbrains. This distribution again suggests that TGF-β3 is not critical in the adult midbrain. Intracellular Smad3 is evident in midbrain dopaminergic neurons, primarily in the cytoplasm, although it has also been detected in the nucleus. Smad3 is also expressed in the ST and in nigrostriatal astrocytes [109,110].

Smad3 deficiency has provided an interesting model of PD [4], with Smad3 deficient mice developing α-synuclein aggregates, and displaying dopaminergic and hippocampal dysfunction. Postnatal neurodegeneration of dopaminergic SN neurons is detectable in these mice, associated to a strong catabolism of striatal DA mediated by monoamine oxidase (MAO) and catechol-*O*-methyltransferase (COMT) enzymes, along with enhanced oxidative stress, and weaker trophic and astrocytic support to dopaminergic neurons. Indeed, α-synuclein inclusions are observed in selected brain areas, which could match areas of the human brain where Lewy bodies are present in PD patients: the SN, paralemniscal nucleus, motor and cingulate cortex, striatum, corpus callosum and spinal cord. These α-synuclein deposits are ubiquitinylated and Ser129 phosphorylated, with a core/halo configuration that resembles the morphology of human Lewy bodies [110]. Other studies have shown that α-synuclein oligomers, a neurotoxic form of α-synuclein found in PD, induce striatal TGF-β1 secretion by reactive astrocytes in order to protect them from neurotoxicity, further evidence of an interaction between TGF-β/Smad3 and α-synuclein dysfunction [118].

Recent studies have focused on the conditional targeting of TGF-β, such as the overexpression of a truncated kinase-defective TβRII under the control of CamKII-tetacycline promoter to inhibit TGF-β signalling. These mice display gait deficits in the footprint assay and mild degeneration of midbrain dopaminergic neurons. By contrast, overexpression of the type I ALK5 receptor through AAV-ALK5 viral injections dampens dopaminergic neurodegeneration and motor deficits after induction of parkinsonism with MPTP [115]. A conditional mutant mouse with DAT-iCre has also been used to selectively remove the TβRII receptor in mature dopaminergic neurons [119]. In these mice no dopaminergic neurodegeneration was observed, although no evaluation of the rostro-caudal distribution was performed, as was the case for a Smad3 deficiency [110,120]. However, this deficit is conditioned to dopaminergic neurons, whereas in the Smad3 knockout mice astrocyte deficiencies could also influence neurodegeneration and α-synuclein aggregation [110]. However, TβRII deficiency in DAT expressing neurons causes a significant reduction in dopaminergic axons, reaching the striatum and the dendrites in the SN [119]. Although the studies on modulation of TGF-β receptors did not investigate α-synuclein alterations as in Smad3 deficient mice, overall the data suggest that through Smad3, TGF-β signalling plays a central role in DA metabolism, neuronal survival and α-synuclein aggregation.

Another possible influence of TGF-β/Smad3 signalling in PD is through the regulation of miRs, although this is yet to be experimentally evaluated. These regulatory elements modulate gene expression, and several of them have been implicated in the aetiology of PD [121]. In particular, miR-205 directly regulates LRRK2 expression, a protein that when mutated may cause or represent a risk factor of familial PD or idiopathic PD, respectively. Downregulation of miR-205 could participate in the increase in LRRK2 observed in the frontal cortex of patients with PD or PD with dementia [122]. TGF-β1 downregulates miR-205 in other systems [79], and a possible interaction in PD would be interesting to investigate.

### 5.2. TGF-β/Smad3 Signalling in Cognition

Cognitive impairment is observed in about 30–40 % of newly diagnosed PD patients with motor symptoms [123,124], which can develop further into dementia as the disease progresses [125]. The molecular and cellular mechanisms driving cognitive impairment in PD patients are currently unknown. TGFβ and Smad3 signalling may be involved, particularly as Smad3 deficiency abolishes hippocampal long-term potentiation (LTP) induction and strongly diminishes the formation of new neurons in the dentate gyrus (DG) of adult mice [120,126]. Smad3 is expressed strongly by mature granule neurons in the hippocampus, although it does not affect their survival. Smad3 is also expressed in neurons newborn in adulthood, which arise in the subgranular zone of the DG, at different stages of differentiation, from late phases of type 2 intermediate progenitor cells to mature granular neurons. Studies using Smad3 null mice have shown a strong decrease in this hippocampal adult neurogenesis, activating apoptosis of type 2 intermediate progenitor cells at the G_1/_S checkpoint of the cell cycle. Hippocampal adult neurogenesis is thought to be implicated in pattern separation, a process that may be involved in the formation of new memories, with no interference with old memories [127]. Indeed, PD patients have impaired adult neurogenesis, which could contribute to non-motor symptoms of the disease, such as cognitive decline and depression [128,129].

Early studies in *Drosophila* and *Aplysia* have shown a role for TGF-β in neuronal plasticity [130,131,132]. TGF-β1 treatment enhances LTP by increasing cAMP response element-binding protein (CREB) phosphorylation [133,134,135], a transcription factor involved in late-LTP and long-term memory [136]. Inhibition of the ALK5 type I receptor with SB431542 decreases late-LTP in the CA1 region of the hippocampus through the phosphorylation of Smad2 and CREB [135]. Applying exogenous TGF-β1 does not affect short-term plasticity in the CA1 [137], and hence, TGF-β1 appears to be involved in the transition from early-phase-LTP into late-phase-LTP in the CA1 through the CREB-mediated transcription of new proteins. However, LTP in the CA1 is not altered in Smad3 null mice, yet it is completely abolished in the DG [120]. Indeed, another member of the TGF-β family, activin, is required for late-LTP and consolidation of long-term memory in the CA1 [138], although some of the roles of activin are independent of Smad signalling but dependent on Erk, PKC or PKA signalling [139]. Behavioural studies have shown that inhibition of the ALK5 type I receptor with SB431542 disrupts memory processes in the object recognition test [135] and in the step-through passive avoidance test [140]. Conditional overexpression of a truncated TβRII under the control of a CaMKII-tet promoter to inhibit TGF-β signalling produces moderate impairment in the Morris water maze [115]. Overall, TGFβ signalling appears to play a central role in the synaptic and cellular plasticity that governs learning and memory processes.

As previously mentioned, Akt activation is a central regulator of TGF-β responsiveness by controlling receptor trafficking to the cell membrane. TGF-β also induces Akt activation, forming a positive feedback loop that amplifies TGF-β signalling [77]. This mechanism may be relevant in diabetes associated with hyperglycaemia, commonly treated with insulin. There is growing evidence that type-2 diabetes and defective insulin signalling may participate in the development of PD [141]. If the positive feedback between Akt and TGF-β is present in the neuronal systems altered in PD, it is possible that defective Akt activation by insulin resistance may reduce TβRI and TβRII exposure at the cell surface, leading to deficient TGF-β/Smad3 signalling and the pathological signs related to parkinsonism. Indeed, the prevalence of cognitive deficits in PD patients with diabetes is higher than in those without diabetes [142], and hippocampal neurons are particularly sensitive to alterations to insulin [143]. Indeed, Akt signalling is involved in the effects of insulin on cognition, which may alter the balance between LTP and LTD (long term depression) [144].

## 6. TGF-β Signalling Modulates GABA Neurotransmission

As described above, cellular and synaptic plasticity in the hippocampal DG is compromised by Smad3 deficiency, significantly limiting adult neurogenesis and abolishing LTP formation. Both processes may be related to GABA neurotransmission, which can regulate proliferation, differentiation, maturation and functional integration of newborn neurons in the DG [127]. Newborn granule neurons are tonically activated by ambient GABA, exerting an excitatory effect through GABA_A_Rs in the immature neuron, as occurs in neonates before the onset of phasic/synaptic activity. In this way, newborn neurons may be modulated by local ambient GABA levels, and hence, by the physiological and pathological conditions in the hippocampus [145]. GABA-mediated excitation in these newborn neurons activates CREB [146], which is also required for LTP to develop in the hippocampus. Indeed, exposing cultured hippocampal neurons to TGF-β2 leads to CREB phosphorylation and a modulation of synaptic plasticity [134]. Moreover, TGF-β2 is required in the developmental shift from excitatory to inhibitory GABA transmission in immature to mature neurons, in which the neuron-specific K^+^-C^l-^ co-transporter KCC2 plays a central role. TGF-β2 can activate trafficking of KCC2 to the membrane to mediate the Cl^–^ extrusion required for the ontogenic change of the GABA response from excitatory/depolarizing to inhibitory/hyperpolarizing conditions. This TGF-β2 effect is mediated by CREB phosphorylation and Rab11b, a molecule involved in vesicular trafficking (Figure 2) [147].

LTP is completely abolished in the hippocampal DG of the Smad3 deficient mouse, but not in the CA1 region [120]. High-frequency synaptic stimulation to the medial perforant path of Smad3-deficient mice does not evoke LTP. This LTP inhibition seems not to be mediated by NMDA or AMPA receptors, but rather by enhanced phasic and tonic GABA_A_R-mediated transmission, promoting an imbalance between excitatory and inhibitory neurotransmission towards inhibition in Smad3 deficiency. In terms of the synaptic properties of granule neurons in the DG, Smad3 deficient mice have a similar resting membrane potential and excitatory postsynaptic potential (EPSP), yet a higher IPSP. Indeed, a higher threshold of action potential firing is found in Smad3 deficient neurons [126]. LTP is more difficult to induce in the granule neurons of the DG than in pyramidal neurons of the CA1, as they are under stronger inhibitory control [148,149]. Indeed, these studies show that Smad3 modulates this inhibitory control. The enhanced GABA neurotransmission observed in Smad3 deficiency seems not to be mediated by increased GABA biosynthesis, since Smad3 deficiency does not alter the levels of the GABA-synthesizing enzymes GAD65 and GAD67 [126]. Alternatively, there may be a decrease in GABA uptake from the extracellular space, stronger GABA release or an increase in the GABA_A_R available. Smad3-deficient mice have fewer astrocytes in different brain areas [110], and it is possible that astrocytic GABA uptake through GATs may be reduced, increasing the ambient GABA. GABA release could also be increased by Smad3 deficiency. However, no alterations in GABA_B_R have been detected, as would be expected for an increment in extracellular GABA. Thus, the third possibility of an increase in the number of GABA_A_Rs seems more likely. TGF-β1 can promote the expression of the α6 GABA_A_R subunit in cerebellar granule neurons [150], and similarly, Smad3 signalling could modulate the expression of a GABA_A_R subunit in the DG. Interestingly, LTP inhibition by Smad3 deficiency may be completely rescued by blocking GABA_A_R with picrotoxin, suggesting that treatment with a GABA_A_R antagonist may be a potential therapy for cognitive impairment in PD [126]. Indeed, increased GABA-mediated inhibition is also seen in the Ts65Dn mouse model of Down’s syndrome, and inhibition of GABA_A_R transmission improves learning and memory deficits in mouse models of Down’s syndrome, AD, Rett’s syndrome and neurofibromatosis [151,152,153,154].

TGF-β signalling in dopaminergic neurons of the SN has further implications related to GABA neurotransmission. TGF-β1 promotes the growth of axons and dendrites in midbrain DAergic neurons, and it is required for the excitatory-inhibitory balance of GABAergic synapses. Like Smad3 deficiency in granular neurons of the hippocampal DG, mice deficient in TβRII receptors in DAergic neurons increase GABAergic inhibitory input in DAergic neurons, diminishing their phasic firing patterns. TβRII deficiency in SN DAergic neurons increases the ratio of inhibitory versus excitatory synapses, and the miniature IPSC (mIPSC) frequencies, and it reduces burst firing of action potentials [119]. GABAergic neurons in the SNpr also express KCC2 to regulate chloride conductance [58], and it would be interesting to evaluate whether deficiencies in TGF-β/Smad3 signalling enhance the inhibitory GABA response in the DG and SN by altering KCC2 trafficking to the membrane of mature neurons.

In addition, the information available in the literature allows us to propose several hypotheses regarding the possible interactions between TGF-β/Smad3 signalling and GABA neurotransmission in relation to PD. For example, phosphorylation of the scaffold protein gephyrin at multiple sites by Erk1/2 and GSK3β promotes changes in gephyrin clustering and influence mIPSCs. Erk1/2 and GSK3β cooperate with calpain to phosphorylate and negatively modulate gephyrin clustering in order to diminish mIPSCs. Alternatively, the inhibition of such phosphorylation may augment mIPSCs and the strength of GABAergic neurotransmission [25]. Smad3 deficient mice have limited Erk1/2 phosphorylation in the dopaminergic neurons of the SN [110], which might reduce gephyrin phosphorylation and promote increased mIPSCs, as observed in the DG of these mice [126]. Another possible mechanism might involve the phosphatases that modulate both GABA_A_Rs and TGF-β/Smad3 signalling, such as PP2A. This phosphatase interacts with PRIP to dephosphorylate the β3 subunits of GABA_A_Rs, and Smad3 in hypoxic conditions [155]. Conversely, the kinases implicated in GABA_A_R phosphorylation, such as PKC and PKA, may also be involved in TGF-β/Smad3 signalling [156].

Finally, disturbing the normal distribution of gephyrin and an abnormal gephyrin accumulation has been observed in AD patients, co-localization with β-amyloid plaques specifically. These gephyrin modifications may affect GABA neurotransmission [157]. It is known that half of the patients with PD and dementia develop β-amyloid plaques and tau-containing neurofibrillary tangles, as well as α-synuclein aggregates, conferring a worse prognosis [158]. TGF-β levels are higher in PD and AD patients, which is associated with the cerebrovascular pathology of the disorder [159]. Indeed, deficiencies in TGF-β1 signalling are associated with increased Aβ deposition and neurofibrillary tangle formation [160], similar to the induction of α-synuclein aggregation in Smad3 deficient mice [110]. We can envision TGF-β signalling deficiency promoting both α-synuclein aggregation and Aβ deposition in PD with dementia.

The interaction between TGF-β/Smad3 signalling and GABA may provide new and interesting insights into the molecular mechanisms involved in PD, PD with dementia or even AD, possibly opening new therapeutic strategies for these disorders.

## 7. Treatment of PD With GABA Modulators

Striatal DA depletion increases the excitatory activity of the STN, and STN ablation ameliorates PD symptoms [161]. Early preclinical studies evaluated the delivery of GABA directly to the STN, and infusion of the GABA_A_R agonist muscimol into the STN and the GPi, in MPTP-treated monkeys, observing that these treatments mitigates STN motor symptoms, such as hyperactivity, akinesia and bradykinesia [162]. Gene therapy approaches to overexpressing GAD in the STN are an interesting alternative [163], and clinical trials with AAV2-GAD viral vectors show improved motor symptoms in PD patients 12 months after viral injection [164].

Considering the enhanced GABA neurotransmission in the hippocampus and SN in TGF-β/Smad3 deficiency, the administration of a GABA_A_R antagonist could be of interest. In this sense, picrotoxin treatment of Smad3 deficient mice completely rescues LTP induction in the DG [126]. Indeed, two studies have shown that flumazenil treatment in PD patients can improve bradykinesia and rigidity [165]. However, while flumazenil is well tolerated, this GABA_A_R antagonist has a short half-life. Its administration is intravenous, and its clinical effects are only evident for 30–60 min after drug administration. Subcutaneous flumazenil infusions may overcome this limitation, providing a new and interesting drug for PD treatment [166]. Furthermore, treatment with GABA modulators could control the activity of SNpr and striatonigral GABA release, which is altered in the dyskinesias that are mediated by L-Dopa treatment [65]. Overall, these studies suggest that therapeutic treatment with GABA modulators may be useful to combat the cognitive and motor deficits associated with PD.

## Abbreviations

AD	Alzheimer’s disease
ATG	Autophagy-related gene
ATP	Adenosine triphosphate
BGT1	Betaine-GABA transporter
CNS	Central Nervous System
COMT	Catechol-*O*-methyltransferase
CREB	cAMP response element-binding protein
DA	Dopamine
DG	Dentate gyrus
DLB	Dementia with Lewy bodies
ECM	Extracellular matrix
GABA	γ-Amino-butyric acid
GABARs	GABA receptors
GABA_A_R	GABA_A_ receptor
GABA_B_R	GABA_B_ receptor
GABARAP	GABA receptor-associated protein
GAD	Glutamic acid decarboxylase
GAT	GABA transporter
GP	Globus pallidus
GPe	external globus pallidus
GPi	internal globus pallidus
GODZ	Golgi-specific DHHC (Asp-His-His-Cys) zinc finger protein
IPSP	Inhibitory postsynaptic potential
LAP	Latent associated protein
LTP	Long term potentiation
MAO	Monoamine oxidase
miRs	microRNAs
MSNs	Medium-sized spiny neurons
NAcc	Nucleus accumbens
NO	Nitric Oxide
NOS	Nitric oxide synthase
PAELR	Parkin-associated endothelin-like receptor
PD	Parkinson’s disease
PSD	Postsynaptic density
SARA	Smad anchor for receptor activation
SN	Substantia nigra
SNpr	Substantia nigra pars reticulata
SNpc	Substantia nigra pars compacta
ST	Striatum
STN	Subthalamic nucleus
TCA	Tricarboxylic acid
TGF-β	Transforming Growth Factor β
Trak1	Trafficking kinesin binding 1 protein
VGAT	vesicular GABA transporter
VTA	ventral tegmental area
